# Supplementary material for: Novel association between TGFA, TGFB1, IRF1, PTGS2 and IKBKB single-nucleotide polymorphisms and occurrence, severity and treatment response of major depressive disorder
Source: PeerJ. 2020 Feb 25;8:e8676. doi: 10.7717/peerj.8676 (PMC7047865; doi:10.7717/peerj.8676)
Supplement: Supplemental Information 1 — * ‘adjusted OR’ means OR adjusted for sex and age; for significant comparisons the superscript b means the bootstrap-boosted OR (resampling with replacement, 10000 iterations); all OR values without bootstrap analysis were calculated using cross-validation algorithm. Statistical power (1 − β) (calculated at α = 0.05) for significant comparisons given in superscripts. p < 0.05 along with corresponding ORs are in bold [file peerj-08-8676-s001.docx]

|  | **Male** | | | |  |  |  |  |
| --- | --- | --- | --- | --- | --- | --- | --- | --- |
| **Genotype**  **/Allele** | **Control** | | **Depression** | | **Crude OR (95% CI)** | ***p*** | **Adjusted OR (95% CI)*** | ***p*** |
|  | **Number** | **Frequency** | **Number** | **Frequency** |  |  |  |  |
| g.41354391A>G of *TGFB1* (rs1800469) | | | | | | | | |
| A/A | 11 | 0.110 | 10 | 0.109 | 0.987 (0.395 -2.459) | 0.977 | 0.967 (0.362-2.580) | 0.946 |
| A/G | 42 | 0.420 | 42 | 0.457 | 1.160 (0.653 -2.060) | 0.610 | 1.136 (0.619-2.087) | 0.680 |
| G/G | 47 | 0.470 | 40 | 0.435 | 0.867 (0.498-1.538) | 0.624 | 0.892 (0.487-1.634) | 0.712 |
| χ2 = 0.278; *p =* 0.870 | | | | | | | | |
| A | 64 | 0.320 | 62 | 0.337 | 1.159 (0.764-1.759) | 0.488 | 1.087 (0.706-1.673) | 0.704 |
| G | 136 | 0.680 | 122 | 0.663 | 0.863 (0.568-1.310) | 0.488 | 0.920 (0.598-1.146) | 0.704 |
| g.70677994G>A of *TGFA* (rs2166975) | | | | | | | | |
| A/A | 13 | 0.126 | 8 | 0.085 | 0.644 (0.254-1.641) | 0.353 | 0.808 (0.302-2.164) | 0.671 |
| **A/G** | **34** | **0.330** | **45** | **0.479** | ***^b^*1.843 (1.037-3.272)**  **1.864  (1.047-3.317)^0.468^** | **0.037**  **0.034** | ***^b^*2.318 (1.222-4.400) 2.280  (1.218-4.268)^0.733^** | **0.010 0.009** |
| G/G | 56 | 0.544 | 41 | 0.436 | 0.649 (0.370-1.145) | 0.132 | ***^b^*0.476 (0.250-0.905) 0.480  (0.257-0.898)^0.740^** | **0.024 0.022** |
| χ2 =4.640; *p =* 0.098 | | | | | | | | |
| A | 60 | 0.291 | 61 | 0.709 | ***^b^*0.109 (0.062-0.189) 0.113  (0.065-0.195)^0.992^** | **<0.001**  **<0.001** | ***^b^*0.106 (0.060-0.186) 0.109  (0.063-0.190)^0.999^** | **<0.001**  **<0.001** |
| G | 146 | 0.324 | 127 | 0.676 | ***^b^*9.005 (5.242-15.468) 8.861  (5.125-15.319)^0.992^** | **<0.001**  **<0.001** | ***^b^*9.281 (5.308-16.225) 9.135  (5.260-15.867)^0.999^** | **<0.001**  **<0.001** |
| g.132484229C>A of *IRF1* (rs2070729) | | | | | | | | |
| A/A | 17 | 0.179 | 16 | 0.170 | 0.941 (0.442-2.005) | 0.874 | 1.197 (0.541-2.650) | 0.658 |
| A/C | 44 | 0.463 | 51 | 0.543 | 1.375 (0.773-2.445) | 0.276 | 1.195 (0.646-2.208) | 0.571 |
| C/C | 34 | 0.358 | 27 | 0.287 | 0.723 (0.370-1.340) | 0.230 | 0.724 (0.376-1.395) | 0.335 |
| χ2 =1.344; *p =* 0.511 | | | | | | | | |
| A | 78 | 0.411 | 83 | 0.441 | 1.139 (0.753-1.724) | 0.537 | 1.157 (0.765-1.749) | 0.490 |
| C | 112 | 0.589 | 105 | 0.559 | 0.878 (0.580-1.328) | 0.537 | 0.865 (0.572-1.307) | 0.490 |
| g.42140549G>T of *IKBKB* (rs5029748) | | | | | | | | |
| G/G | 61 | 0.610 | 53 | 0.589 | 0.916 (0.510-1.644) | 0.769 | 0.955 (0.514-1.776) | 0.885 |
| **G/T** | **19** | **0.190** | **30** | **0.333** | ***^b^*2.153 (1.082-4.288) 2.132  (1.097-4.143)^0.466^** | **0.029**  **0.026** | ***^b^*2.073 (1.016-4.300)**  **2.063  (1.024-4.154)^0.423^** | **0.049**  **0.049** |
| **T/T** | **20** | **0.200** | **7** | **0.078** | ***^b^*0.316 (0.118-0.849)**  **0.337  (0.135-0.841)^0.758^** | **0.022**  **0.020** | ***^b^*0.295 (0.100-0.869)**  ***^cv^*0.310  (0.116-0.830)^0.799^** | **0.027**  **0.021** |
| χ2 =8.788; *p =* 0.012 | | | | | | | | |
| G | 141 | 0.705 | 136 | 0.756 | 1.211 (0.817-1.796) | 0.341 | 1.305 (0.826-2.061) | 0.253 |
| T | 59 | 0.295 | 44 | 0.244 | 0.826 (0.557-1.225) | 0.341 | 0.766 (0.485-1.210) | 0.253 |
| g.186643058A>G of *PTGS2* (rs5275) | | | | | | | | |
| **A/A** | **40** | **0.392** | **48** | **0.505** | **1.583 (0.896-2.796)** | **0.111** | ***^b^*2.073 (0.999-4.300)**  **1.803  (0.982-3.309)^0.464^** | **0.050 0.057** |
| A/G | 41 | 0.402 | 37 | 0.389 | 0.949 (0.534-1.687) | 0.858 | 0.852 (0.462-1.575) | 0.611 |
| G/G | 21 | 0.206 | 10 | 0.105 | 0.454 (0.201-1.028) | 0.057 | ***^b^*0.427 (0.171-1.019)**  **0.438 (0.186-1.032)^0.599^** | **0.052 0.059** |
| χ2 =4.593; *p =*0.101 | | | | | | | | |
| **A** | **121** | **0.593** | **133** | **0.700** | ***^b^*1.588 (1.031-2.445)**  **1.601  (1.054-2.430)^0.672^** | **0.036**  **0.027** | ***^b^*1.659 (1.064-2.586)**  **1.664  (1.087-2.548)^0.745^** | **0.025 0.019** |
| **G** | **83** | **0.407** | **57** | **0.300** | ***^b^*0.621 (0.399-0.968)**  **0.625  (0.412-0.949)^0.608^** | **0.035**  **0.027** | ***^b^*0.603 (0.393-0.926)**  **0.601  (0.393-0.920)^0.666^** | **0.021 0.019** |
| g.186640617C>T of *PTGS2* (rs4648308) | | | | | | | | |
| C/C | 65 | 0.663 | 67 | 0.736 | 1.417 (0.754-2.664) | 0.276 | 1.335 (0.687-2.595) | 0.394 |
| C/T | 25 | 0.255 | 24 | 0.264 | 1.046 (0.543-2.014) | 0.892 | 1.128 (0.564-2.255) | 0.734 |
| T/T | 8 | 0.082 | 0 | 0 | **-** | - | - | - |
| χ2 =7.802; *p =* 0.020 | | | | | | | | |
| **C** | **155** | **0.791** | **158** | **0.868** | ***^b^*1.772 (0.996-3.162)**  **1.741  (1.004-3.019)^0.848^** | **0.052**  **0.048** | ***^b^*1.744 (0.983-3.094)**  **1.751  (1.007-3.040)^0.854^** | **0.049 0.047** |
| **T** | **41** | **0.209** | **24** | **0.132** | ***^b^*0.567 (0.322-0.996)**  **0.574  (0.331-0.996)^0.553^** | **0.049**  **0.048** | ***^b^*0.566 (0.315-0.999)**  **0.571  (0.329-0.993)^0.558^** | **0.049 0.047** |
|  | **Female** | | | |  |  |  |  |
| **Genotype**  **/Allele** | **Control** | | **Depression** | | **Crude OR (95% CI)** | ***p*** | **Adjusted OR (95% CI)*** | ***p*** |
|  | **Number** | **Frequency** | **Number** | **Frequency** |  |  |  |  |
| g.41354391A>G of *TGFB1* (rs1800469) | | | | | | | | |
| A/A | 12 | 0.150 | 10 | 0.114 | 0.726 (0.293 -1.799) | 0.487 | 0.556 (0.204-1.524) | 0.255 |
| A/G | 29 | 0.363 | 34 | 0.386 | 1.107 (0.589 -2.080) | 0.750 | 1.286 (0.654-2.529) | 0.466 |
| G/G | 39 | 0.488 | 44 | 0.500 | 1.051 (0.571-1.935) | 0.871 | 1.016 (0.529-1.953) | 0.962 |
| χ2 =0.500 ; *p =* 0.779 | | | | | | | | |
| A | 53 | 0.331 | 54 | 0.307 | 0.893 (0.581-1.373) | 0.606 | 0.857 (0.522-1.407) | 0.542 |
| G | 107 | 0.669 | 122 | 0.693 | 1.069 (0.698-1.637) | 0.606 | 1.167 (0.711-1.917) | 0.542 |
| g.70677994G>A of *TGFA* (rs2166975) | | | | | | | | |
| A/A | 14 | 0.161 | 7 | 0.076 | 0.429 (0.163-1.129) | 0.084 | 0.399 (0.138-1.147) | 0.088 |
| A/G | 25 | 0.287 | 38 | 0.413 | 1.745 (0.932-3.267) | 0.080 | 1.893 (0.968-3.701) | 0.062 |
| G/G | 48 | 0.552 | 47 | 0.511 | 0.849 (0.470-1.534) | 0.584 | 0.796 (0.422-1.502) | 0.482 |
| χ2 =4.891; *p =* 0.087 | | | | | | | | |
| A | 53 | 0.305 | 52 | 0.283 | 0.912 (0.596-1.394) | 0.670 | 0.926 (0.567-1.514) | 0.760 |
| G | 121 | 0.695 | 132 | 0.717 | 1.097 (0.717-1.677) | 0.670 | 1.081 (0.661-1.765) | 0.760 |
| g.132484229C>A of *IRF1* (rs2070729) | | | | | | | | |
| A/A | 20 | 0.244 | 20 | 0.215 | 0.849 (0.417-1.730) | 0.650 | 0.655 (0.297-1.437) | 0.291 |
| **A/C** | **32** | **0.390** | **48** | **0.516** | **1.667 (0.910-3.056)** | **0.096** | ***^b^*2.016 (1.025-3.966)**  **1.936  (1.003-3.738)^0.508^** | **0.042 0.049** |
| C/C | 30 | 0.366 | 25 | 0.269 | 0.637 (0.334-1.216) | 0.169 | 0.657 (0.328-1.318) | 0.237 |
| χ2 =2.975; *p =*0.226 | | | | | | | | |
| A | 72 | 0.439 | 88 | 0.473 | 1.136 (0.756-1.706) | 0.539 | 1.159 (0.730-1.840) | 0.532 |
| C | 92 | 0.561 | 98 | 0.527 | 0.880 (0.586-1.322) | 0.539 | 0.862 (0.544-1.370) | 0.532 |
| g.42140549G>T of *IKBKB* (rs5029748) | | | | | | | | |
| G/G | 47 | 0.560 | 47 | 0.528 | 0.881 (0.482-1.610) | 0.678 | 0.899 (0.473-1.709) | 0.746 |
| G/T | 21 | 0.250 | 29 | 0.326 | 1.450 (0.743-2.829) | 0.273 | 1.521 (0.749-3.089) | 0.246 |
| T/T | 16 | 0.190 | 13 | 0.146 | 0.731 (0.324-1.631) | 0.436 | 0.644 (0.269-1.539) | 0.323 |
| χ2 =1.447; *p =* 0.485 | | | | | | | | |
| G | 115 | 0.685 | 123 | 0.691 | 1.080 (0.733-1.591) | 0.698 | 1.082 (0.664-1.764) | 0.751 |
| T | 53 | 0.315 | 55 | 0.309 | 0.979 (0.662-1.449) | 0.698 | 0.924 (0.567-1.506) | 0.751 |
| g.186643058A>G of *PTGS2* (rs5275) | | | | | | | | |
| A/A | 39 | 0.459 | 33 | 0.359 | 0.661 (0.359-1.211) | 0.176 | 0.595 (0.309-1.143) | 0.119 |
| **A/G** | **34** | **0.400** | **46** | **0.500** | **1.500 (0.823-2.734)** | **0.183** | **1.962 (1.024-3.758) 1.952  (1.017-3.746)^0.524^** | **0.042**  **0.044** |
| G/G | 12 | 0.141 | 13 | 0.141 | 1.001 (0.427-2.344) | 0.998 | 0.718 (0.284-1.812) | 0.483 |
| χ2 =2.006; *p =* 0.356 | | | | | | | | |
| A | 112 | 0.659 | 112 | 0.609 | 0.810 (0.527-1.244) | 0.336 | 0.833 (0.524-1.325) | 0.441 |
| G | 58 | 0.341 | 72 | 0.391 | 1.235 (0.804-1.898) | 0.336 | 0.816 (0.755-1.908) | 0.441 |
| g.186640617C>T of *PTGS2* (rs4648308) | | | | | | | | |
| C/C | 65 | 0.756 | 57 | 0.655 | 0.614 (0.315-1.195) | 0.148 | 0.595 (0.294-1.205) | 0.149 |
| **C/T** | **15** | **0.174** | **28** | **0.322** | ***^b^*2.270 (1.100-4.684)**  **2.246  (1.098-4.596)^0.806^** | **0.027**  **0.027** | ***^b^*2.574 (1.224-5.415)**  **2.533  (1.178-5.449)^0.587^** | **0.013 0.017** |
| T/T | 6 | 0.070 | 2 | 0.023 | 0.314 (0.061-1.620) | 0.163 | 0.211 (0.037-1.211) | 0.081 |
| χ2 =6.449; *p =* 0.039 | | | | | | | | |
| C | 145 | 0.843 | 142 | 0.816 | 0.843 (0.495-1.435) | 0.530 | 0.853 (0.469-1.553) | 0.603 |
| T | 27 | 0.157 | 32 | 0.184 | 1.186 (0.697-2.018) | 0.530 | 1.172 (0.644-2.135) | 0.603 |
