# Supplementary material for: Novel association between TGFA, TGFB1, IRF1, PTGS2 and IKBKB single-nucleotide polymorphisms and occurrence, severity and treatment response of major depressive disorder
Source: PeerJ. 2020 Feb 25;8:e8676. doi: 10.7717/peerj.8676 (PMC7047865; doi:10.7717/peerj.8676)
Supplement: Supplemental Information 2 — * ‘adjusted OR’ means OR adjusted for sex and age; for significant comparisons the superscript b means the bootstrap-boosted OR (resampling with replacement, 10,000 iterations); all OR values without bootstrap analysis were calculated using cross-validation algorithm. Statistical power (1 − β) (calculated at α = 0.05) for significant comparisons given in superscripts. p < 0.05 along with corresponding ORs are in bold [file peerj-08-8676-s002.docx]

| **Combined genotype** | **Control** | | **Depression (n=180)** | | **Crude OR (95% CI)** | ***p*** | **Adjusted OR (95% CI)** | ***p*** |
| --- | --- | --- | --- | --- | --- | --- | --- | --- |
|  | **Number** | **Frequency** | **Number** | **Frequency** |  |  |  |  |
| g.41354391A>G of *TGFB1* (rs1800469) - g.132484229C>A of *IRF1* (rs2070729) | | | | | | | | |
| A/A-A/A | 4 | 0.023 | 2 | 0.011 | 0.477 (0.086-3.658) | 0.397 | 0.441 (0.068-2.841) | 0.389 |
| A/A-A/C | 9 | 0.052 | 9 | 0.050 | 0.965 (0. 372-2.499) | 0.941 | 0.747 (0.258-2.167) | 0.592 |
| A/A-C/C | 8 | 0.046 | 9 | 0.050 | 1.092 (0.410-2.908) | 0.860 | 1.123 (0.386-3.266) | 0.831 |
| G/G -A/A | 20 | 0.116 | 17 | 0.095 | 0.803 (0.404-1.594) | 0.529 | 0.664 (0.312-1.411) | 0.287 |
| G/G-A/C | 37 | 0.214 | 43 | 0.240 | 1.162 (0.704-1.919) | 0.556 | 1.223 (0.713-2.099) | 0.465 |
| G/G-C/C | 27 | 0.156 | 24 | 0.134 | 0.837 (0.462-1.517) | 0.558 | 0.846 (0.452-1.584) | 0.601 |
| A/G-A/A | 11 | 0.064 | 16 | 0.089 | 1.446 (0.649-3.220) | 0.365 | 1.615 (0.698-3.737) | 0.263 |
| A/G-C/C | 28 | 0.162 | 18 | 0.101 | 0. 579 (0.307-1.093) | 0.091 | 0.614 (0.309-1.219) | 0.163 |
| A/G-A/C | 29 | 0.168 | 41 | 0.229 | 1.147 (0.867-2.511) | 0.150 | 1.485 (0.842-2.618) | 0.172 |
| g.41354391A>G of *TGFB1* (rs1800469) - g.70677994G>A of *TGFA* (rs2166975) | | | | | | | | |
| A/A-A/A | 4 | 0.024 | 1 | 0.005 | 0.250 (0.027-2.279) | 0.217 | 0.192 (0.018-2.011) | 0.168  0.522113  0. 941 |
| A/A-A/G | 6 | 0.031 | 8 | 0.043 | 1.370 (0.465-44.042 | 0.567 | 1.443 (0.469-4.438) | 0.522  0.860 |
| A/A-G/G | 13 | 0.068 | 11 | 0.059 | 0.851 (0.370-1.956) | 0.703 | 0.661 (0.259-1.687) | 0.386 |
| G/G-A/A | 12 | 0.063 | 8 | 0.043 | 0.663 (0.264-1.666) | 0.380 | 0.643 (0.236-1.757) | 0.389  0.556 |
| G/G-A/G | 28 | 0.147 | 33 | 0.176 | 1.239 (0.714-2.151) | 0.444 | 1.311 (0.733-2.347) | 0.361  0.558 |
| G/G-G/G | 45 | 0.236 | 42 | 0.223 | 0.933 (0.577-1.509) | 0.778 | 0.873 (0.522-1.463) | 0.606  0.365 |
| A/G-A/A | 8 | 0.042 | 6 | 0.032 | 0.754 (0.256-2.224) | 0.608 | 0.927 (0.297-2.887) | 0.895  0.091 |
| A/G -G/G | 39 | 0.204 | 34 | 0.181 | 0.860 (0.515-1.438) | 0.565 | 0.779 (0.450-1.347) | 0.371  0.150 |
| **A/G-A/G** | **24** | **0.126** | **35** | **0.186** | **1.592 (0.904-2.803)** | **0.106** | ***^b^*1.906 (1.032-3.518)**  ***^cv^*1.898  (1.036-3.477)^0.490^** | **0.039**  **0.038** |
| g.41354391A>G of *TGFB1* (rs1800469) - g.186643058A>G of *PTGS2* (rs5275) | | | | | | | | |
| A/A-A/A | 7 | 0.037 | 8 | 0.043 | 1.168 (0.414-3. 300) | 0.768 | 1.196 (0.391-3.664) | 0.754  0. 941 |
| A/A-A/G | 12 | 0.063 | 8 | 0.043 | 0.663 (0.264-1.666) | 0.380 | 0.643 (0.235-1.761) | 0.390  0.860 |
| A/A-G/G | 4 | 0.021 | 4 | 0.021 | 1.016(0.249-4.143) | 0.982 | 0.539 (0.117-2.479) | 0.427  0.529 |
| G/G-A/A | 41 | 0.215 | 33 | 0.176 | 0.779 (0.467-1.300) | 0.337 | 0.817 (0.473-1.410) | 0.468  0.556 |
| G/G-A/G | 30 | 0.157 | 39 | 0.207 | 1. 405 (0.829-2.380) | 0.205 | 1.367 (0.782-2.391) | 0.273  0.558 |
| G/G-G/G | 14 | 0.073 | 12 | 0.064 | 0.862 (0.387-1.922) | 0. 716 | 0.793 (0.333-1.881) | 0.597  0.365 |
| A/G-A/A | 29 | 0.152 | 37 | 0.197 | 1.369 (0.801-2.340) | 0.249 | 1.381 (0.778-2.445) | 0.270  0.091 |
| A/G -G/G | 10 | 0.052 | 6 | 0.032 | 0.597 (0.212-1.682) | 0.327 | 0.534 (0.181-1.576) | 0.256  0.150 |
| A/G-A/G | 29 | 0.152 | 32 | 0.170 | 1.146 (0.661-1.987) | 0.627 | 1.319 (0.733-2.371) | 0.357  0.150 |
| g.41354391A>G of *TGFB1* (rs1800469) - g.186640617C>T of *PTGS2* (rs4648308) | | | | | | | | |
| A/A-C/C | 14 | 0.073 | 12 | 0.064 | 0.862 (0.387-1.921) | 0.716 | 0.760 (0.319-1.811) | 0.535  0.467  0. 941 |
| A/A-C/T | 7 | 0.037 | 4 | 0.021 | 0.571 (0.164-1. 994) | 0.379 | 0.601 (0.152-2.370) | 0.467  0.860 |
| A/A-T/T | 1 | 0.005 | 1 | 0.005 | 1.016 (0.063-16.510) | 0.991 | 0.351 (0.021-5.995) | 0.469  0.529 |
| G/G-C/C | 64 | 0.335 | 57 | 0.303 | 0.863 (0.560-1.332) | 0.506 | 0.844 (0.532-1.339) | 0.471  0.556 |
| G/G-C/T | 15 | 0.079 | 22 | 0.117 | 1.555 (0.778-3.106) | 0.210 | 1.607 (0.761-3.394) | 0.213  0.558 |
| G/G-T/T | 4 | 0.023 | 0 | 0.000 | - | - | - | -  0.365 |
| A/G-C/C | 49 | 0.257 | 50 | 0.266 | 1.050 (0.663-1.663) | 0.835 | 1.078 (0.661-1.759) | 0.763  0.091 |
| A/G-C/T | 17 | 0.089 | 23 | 0.122 | 1.427 (0.734-2.772) | 0.293 | 1.760 (0.873-3.548) | 0.114  0.150 |
| A/G-T/T | 3 | 0.016 | 1 | 0.005 | 0.335 (0.034-3.279) | 0.345 | 0.260 (0.025-2.743) | 0.263  0.150 |
| g.41354391A>G of *TGFB1* (rs1800469) - g.42140549G>T of *IKBKB* (rs5029748) | | | | | | | | |
| A/A-G/G | 13 | 0.068 | 11 | 0.059 | 0.851 (0.370-1.956) | 0.703 | 0.584 (0.229-1.488) | 0.260 |
| A/A-G/T | 2 | 0.010 | 6 | 0.032 | 3.115 (0. 617-15.733) | 0.168 | 3.405 (0.646-17.933) | 0.148  0.860 |
| A/A-T/T | 6 | 0.031 | 1 | 0.005 | 0.165 (0.019-1.397) | 0.0.097 | 0.237 (0.027-2.115) | 0.197  0.529 |
| G/G-G/G | 48 | 0.251 | 35 | 0.186 | 0.682 (0.416-1.116) | 0.126 | 0.696 (0.411-1.180) | 0.178  0.130  0.556 |
| G/G-G/T | 24 | 0.126 | 35 | 0.186 | 1.592 (0.904-2.803) | 0. 106 | 1.588 (0.873-2.887) | 0.130  0.558 |
| G/G-T/T | 11 | 0.058 | 12 | 0.064 | 1.116 (0.478-2.602) | 0.799 | 1.022 (0.406-2.571) | 0.963  0.365 |
| A/G-G/G | 45 | 0.236 | 49 | 0.261 | 1. 144 (0.716-1.144) | 0.573 | 1.255 (0.761-2.069) | 0.373  0.091 |
| A/G-G/T | 13 | 0.068 | 16 | 0.085 | 1.274 (0.593-2.734) | 0.533 | 1.274 (0.571-2.840) | 0.554  0.150 |
| A/G-T/T | 13 | 0.068 | 7 | 0.037 | 0.530 (0.206-1.363) | 0.186 | 0.419 (0.151-1.162) | 0.095  0.150 |
| g.70677994G>A of *TGFA* (rs2166975) – g.132484229C>A of *IRF1* (rs2070729) | | | | | | | | |
| A/A-A/A | 6 | 0.031 | 3 | 0.016 | 0.467 (0.115-1.897)  0.503 (0.123-2.050) | 0.287  0.336 | 0.492 (0.106-2.288) | 0.366  0. 941 |
| A/A-A/C | 12 | 0.063 | 9 | 0.048 | 0.699 (0.287-1.702)  0.754 (0.309-1.841) | 0.43  0.534 | 0.838 (0.318-2.209) | 0.721  0.860 |
| A/A-C/C | 8 | 0.042 | 3 | 0.016 | 0.346 (0.090-1.326)  0.373 (0.097-1.435) | 0.122  0.150 | 0.401 (0.097-1.665) | 0.209  0.529 |
| G/G-A/A | 15 | 0.079 | 15 | 0.080 | 0.947 (0.449-2.000)  1.023 (0.484-2.163) | 0.887  0.952 | 0.917 (0.410-2.050) | 0.833 |
| G/G-A/C | 37 | 0.194 | 44 | 0.235 | 1.172 (0.714-1.925)  1.281 (0.781-2.101) | 0.53  0.325 | 1.156 (0.679-1.969) | 0.593  0.558 |
| G/G-C/C | 39 | 0.204 | 29 | 0.155 | 1.086 (0.525-2.247)  0.653 (0.383-1.112)  0.715 (0.421-1.217) | 0.117  0.215 | 0.680 (0.384-1.204) | 0.186  0.365 |
| A/G-A/A | 15 | 0.079 | 17 | 0.091 | 1.173 (0.567-2.431) | 0.824  0.666 | 1.218 (0.561-2.642) | 0.618  0.091 |
| A/G-C/C | 17 | 0.089 | 20 | 0.107 | 1.134 (0.573-2.243)  1.226 (0.619-2.426) | 0.718  0.558 | 1.408 (0.687-2.886) | 0.349  0.150 |
| **A/G-A/C** | **27** | **0.141** | **45** | **0.241** | ***^b^*1.951 (1.152-3. 305)**  **1.925 (1.136-3.262)^0.554^** | **0.013**  **0.015** | ***^b^*2.117 (1.224-3.660)**  **2.092 (1.193-3.660)^0.667^** | **0.007**  **0.010** |
| g.70677994G>A of *TGFA* (rs2166975)- g.186643058A>G of *PTGS2* (rs5275) | | | | | | | | |
| G/G-A/A | 41 | 0.215 | 38 | 0.202 | 0.914 (0.556-1.503) | 0.724 | 0.790 (0.461-1.354) | 0.391 |
| G/G-A/G | 40 | 0.209 | 41 | 0.218 | 1.039 (0.635-1.701) | 0.878 | 1.048 (0.617-1.778) | 0.863 |
| **G/G-G/G** | **22** | **0.115** | **8** | **0.043** | ***^b^*0.320 (0.127-0.807)**  **0.341 (0.148-0.788)^0.828^** | **0.016**  **0.012** | ***^b^*0.223 0.087 0.574**  **0.233  (0.094-0.579)^0.940^** | **0.002**  **0.002** |
| A/A-A/A | 10 | 0.052 | 7 | 0.037 | 0.692 (0.258-1.859) | 0.465 | 0.960 (0.331-2.784) | 0.940 |
| **A/A-G/G** | **7** | **0.037** | **1** | **0.005** | **0.139 (0.017-1.141)** | **0.066** | ***^b^*0.167 (0.027-1.031)**  ***^cv^*0.129 (0.014-1.159)^0.805^** | **0.054**  **0.068** |
| A/A-A/G | 10 | 0.052 | 7 | 0.037 | 0.692 (0.258-1.859) | 0.465 | 0.639 (0.218-1.877) | 0.415 |
| A/G-A/A | 28 | 0.147 | 34 | 0.181 | 1.271 (0.735-2.197) | 0.391 | 1.398 (0.784-2.494) | 0.257 |
| **A/G-G/G** | **4** | **0.021** | **14** | **0.074** | ***^b^*3.581 (1.233-13.12)**  **3.761 (1.215-11.647)^0.291^** | **0.026**  **0.022** | ***^b^*4.264 (1.416-12.839)**  **4.137 (1.263-13.545)^0.291^** | 0.010  0.019 |
| A/G-A/G | 24 | 0.126 | 35 | 0.186 | 1.575 (0.895-2.771) | 0.115  0.106 | 1.756 (0.966-3.193) | 0.065 |
| g.70677994G>A of *TGFA* (rs2166975)- g.186640617C>T of *PTGS2* (rs4648308) | | | | | | | | |
| G/G-C/C | 66 | 0.346 | 61 | 0.324 | 0.940 (0.610-1.450) | 0.781 | 0.758 (0.477-1.203) | 0.240 |
| G/G-C/T | 22 | 0.115 | 22 | 0.117 | 1.045 (0.556-1.965) | 0.89 | 1.048(0.532-2.066) | 0.892 |
| **G/G-T/T** | **12** | **0.063** | **1** | **0.005** | ***^b^*0.087 (0.013-0.638)**  **0.080 (0.010-0.620)^0.942^** | **0.018**  **0.016** | ***^b^*0.057 (0.011-0.312)**  **0.051 (0.006-0.420) ^0.948^** | **0.001**  **0.006** |
| A/A-C/C | 16 | 0.084 | 9 | 0.048 | 0.563 (0.242-1.309) | 0.182 | 0.570 (0.231-1.404) | 0.222 |
| A/A-C/T | 8 | 0.042 | 5 | 0.027 | 0.640 (0.205-1.994) | 0.441 | 0.729 (0.213-2.497) | 0.615 |
| A/A-T/T | 2 | 0.010 | 0 | 0 | - | - | - | - |
| A/G-C/C | 47 | 0.246 | 53 | 0.282 | 1.247 (0.785-1.980) | 0.35 | 1.379 (0.848-2.244) | 0.196 |
| **A/G-C/T** | **10** | **0.052** | **25** | **0.133** | ***^b^*3.005 (1.242-7.269)**  **2.776 (1.294-5.956)^0.584^** | **0.015**  **0.009** | ***^b^*3.240 (1.442-7.280)**  **3.115  (1.397-6.944) ^0.663^** | **0.004**  **0.005** |
| A/G-T/T | 0 | 0 | 0 | 0 | - | - | - | - |
| g.70677994G>A of *TGFA* (rs2166975)- g.42140549G>T of *IKBKB* (rs5029748) | | | | | | | | |
| G/G-G/G | 54 | 0.283 | 46 | 0.246 | 0.839 (0.529-1.331) | 0.456 | 0.720 (0.437-1.186) | 0.197 |
| G/G-G/T | 25 | 0.131 | 29 | 0.154 | 1.238 (0.693-2.210) | 0.471 | 1.125 (0.609-2.077) | 0.708 |
| **G/G-T/T** | **23** | **0.120** | **9** | **0.048** | ***^b^*0.362 (0.156-0.840)**  **0.367  (0.165-0.816)^0.801^** | **0.018**  **0.014** | ***^b^*0.286 (0.106-0.772)**  **0.306  (0.131 0.719^0.882^** | **0.013**  **0.007** |
| A/A-G/G | 13 | 0.068 | 9 | 0.048 | 0.701 (0.292-1.682) | 0.426 | 0.815 (0.320-2.074) | 0.668 |
| A/A-G/T | 4 | 0.021 | 6 | 0.032 | 1.570 (0.435-5.659) | 0.491 | 1.314 (0.323-5.343) | 0.703 |
| A/A-T/T | 7 | 0.037 | 0 | 0 | - | - | - | - |
| A/G-G/G | 41 | 0.215 | 44 | 0.234 | 1.145 (0.704-1.863) | 0.585 | 1.199 (0.717-2.003) | 0.489 |
| **A/G-G/T** | **11** | **0.058** | **24** | **0.128** | ***^b^*2.393 (1.136-5.042)**  **2.395  (1.138-5.041)^0.472^** | **0.022**  **0.021** | ***^b^*2.645 (1.184-5.910)**  **2.621  (1.208-5.688)^0.571^** | **0.018**  **0.015** |
| A/G-T/T | 6 | 0.031 | 11 | 0.059 | 1.954 (0.707-5.402) | 0.197 | 1.923 (0.638-5.799) | 0.246 |
| g.132484229C>A of *IRF1* (rs2070729)- g.186643058A>G of *PTGS2* (rs5275) | | | | | | | | |
| C/C-A/A | 31 | 0.162 | 24 | 0.128 | 0.646 (0.360-1.160) | 0.143 | 0.772 (0.416-1.435) | 0.414 |
| C/C-A/G | 26 | 0.136 | 20 | 0.106 | 0.720 (0.338-1.333) | 0.295 | 0.799 (0.408-1.567) | 0.514 |
| C/C-G/G | 7 | 0.037 | 7 | 0.037 | 0.927 (0.319-2.700) | 0.890 | 0.909 (0.301-2.750) | 0.866 |
| A/A-A/A | 11 | 0.058 | 17 | 0.090 | 1.481 (0.673-3.259) | 0.329 | 1.445 (0.622-3.360) | 0.392 |
| A/A-A/G | 18 | 0.094 | 14 | 0.074 | 0.701 (0.337-1.456) | 0.341 | 0.943 (0.438-2.028) | 0.880 |
| A/A-G/G | 7 | 0.037 | 5 | 0.027 | 0.655 (0.204-2.104) | 0.477 | 0.523 (0.138-1.981) | 0.340 |
| A/C-A/A | 33 | 0.173 | 40 | 0.213 | 1.162 (0.694-1.947) | 0.568 | 1.444 (0.832-2.506) | 0.191 |
| **A/C-A/G** | **29** | **0.152** | **49** | **0.261** | ***^b^*2.077 (1.206-3.576)**  **1.969 (1.180-3.286)^0.614^** | **0.008**  **0.009** | ***^b^*1.863 (1.022-3.394)**  **1.844  (1.069-3.180)^0.515^** | 0.042  0.028 |
| A/C-G/G | 11 | 0.058 | 10 | 0.053 | 0.926 (0.391-2.193) | 0.861 | 0.830 (0.325-2.121) | 0.697 |
| g.132484229C>A of *IRF1* (rs2070729)- g.186640617C>T of *PTGS2* (rs4648308) | | | | | | | | |
| C/C-C/C | 48 | 0.251 | 35 | 0.186 | 0.622 (0.378-1.024) | 0.062 | 0.684 (0.405-1.155) | 0.155 |
| C/C-C/T | 13 | 0.068 | 12 | 0.064 | 0.873 (0.387-1.971) | 0.744 | 0.965 (0.399-2.333) | 0.937 |
| C/C-T/T | 0 | 0 | 0 | 0 | **-** | **-** | - | - |
| A/A-C/C | 26 | 0.136 | 21 | 0.112 | 0.741 (0.399-1.374) | 0.341 | 0.762 (0.395-1.470) | 0.418 |
| A/A-C/T | 8 | 0.042 | 11 | 0.059 | 1.334 (0.523-3.401) | 0.546 | 1.775 (0.663-4.755) | 0.253 |
| A/A-T/T | 2 | 0.010 | 2 | 0.011 | 0.955 (0.133-6.854) | 0.963 | 1.401 (0.163-12.068) | 0.759 |
| A/C-C/C | 53 | 0. 277 | 68 | 0.362 | 1.365 (0.876-2.126) | 0.169 | 1.482 (0.931-2.358) | 0.097 |
| A/C-C/T | 17 | 0.089 | 28 | 0.149 | 1.752 (0.924-3.321) | 0.086 | 1.848 (0.931-3.665) | 0.079 |
| A/C-T/T | 3 | 0.016 | 0 | 0 | - | - | - | - |
| g.132484229C>A of *IRF1* (rs2070729)- g.42140549G>T of *IKBKB* (rs5029748) | | | | | | | | |
| C/C-G/G | 36 | 0. 188 | 26 | 0.138 | 0.641 (0.368-1.118) | 0.117 | 0.800 (0.443-1.442) | 0.457 |
| C/C-G/T | 15 | 0.079 | 18 | 0.096 | 1.170 (0.570-2.403) | 0.669 | 1.025 (0.482-2.181) | 0.949 |
| C/C-T/T | 11 | 0.058 | 6 | 0.032 | 0.507 (0.183-1.404) | 0.191 | 0.523 (0.174-1.574) | 0.249 |
| A/A-G/G | 23 | 0.120 | 20 | 0.106 | 0.815 (0.430-1.544) | 0.530  0 | 0.747 (0.374-1.489) | 0.407 |
| A/A-G/T | 7 | 0.037 | 11 | 0.059 | 1.543 (0.584-4.078) | 0.382 | 2.262 (0.812-6.300) | 0.118 |
| A/A-T/T | 4 | 0.021 | 3 | 0.016 | 0.716 (0.158-3.246) | 0.665 | 0.553 (0.113-2.702) | 0.464 |
| A/C-G/G | 46 | 0.241 | 54 | 0.287 | 1.152 (0.723-1.837) | 0.552 | 1.252 (0.766-2.048) | 0.370 |
| **A/C-G/T** | **16** | **0.084** | **29** | **0.154** | ***^b^*2.032 (1.036-3.989)**  **1.995 (1.044-3.810)^0.402^** | **0.039**  **0.036** | ***^b^*1.918(0.935-3.931)**  **1.901  (0.958-3.774)^0.362^** | 0.075  0.066 |
| A/C-T/T | 13 | 0.068 | 11 | 0.059 | 0.801 (0.348-1.839) | 0.600 | 0.895 (0.363-2.203) | 0.809 |
| g.42140549G>T of *IKBKB* (rs5029748)- g.186643058A>G of *PTGS2* (rs5275) | | | | | | | | |
| G/G-A/A | 43 | 0.225 | 49 | 0.261 | 1.210 (0.753-1.946) | 0.431 | 1.190 (0.718-1.972) | 0.500 |
| G/G-A/G | 48 | 0.251 | 38 | 0.202 | 0.746 (0.458-1.215) | 0.240 | 0.812 (0.483-1.364) | 0.431 |
| G/G-G/G | 14 | 0.073 | 12 | 0.064 | 0.857 (0.385-1.909) | 0.706 | 0.691 (0.289-1.651) | 0.405 |
| T/T-A/A | 13 | 0.068 | 8 | 0.043 | 0.605 (0.244-1.496) | 0.276 | 0.461 (0.172-1.236) | 0.124 |
| T/T-A/G | 9 | 0.047 | 10 | 0.053 | 1.131 (0.448-2.853) | 0.794 | 1.217 (0.443-3.343) | 0.704 |
| **T/T-G/G** | **14** | **0.073** | **2** | **0.011** | ***^b^*0.131 (0.037-0.598)**  **0.136**  **(0.030-0.607)^0.936^** | **0.008**  **0.009** | ***^b^*0.126 (0.027-0.589)**  **0.132  (0.028-0.610)^0.939^** | **0.008**  **0.009** |
| G/T-A/A | 19 | 0. 099 | 21 | 0.112 | 1.133 (0.587-2.189) | 0.709 | 1.331 (0.662-2.678) | 0.422 |
| **G/T-A/G** | **16** | **0.084** | **31** | **0.165** | ***^b^*2.235 (1.114-4.487)**  **2.160  (1.138-4.098)^0.512^** | **0.024**  **0.018** | ***^b^*1.933 (0.883-4.233)**  **1.894  (0.968-3.704)^0.357^** | **0.008**  **0.009** |
| G/T-G/G | 4 | 0.021 | 7 | 0.037 | 1.801 (0.518-6.264) | 0.355 | 1.590 (0.424-5.963) | 0.492 |
| g.42140549G>T of *IKBKB* (rs5029748) – g.186640617C>T of *PTGS2* (rs4648308) | | | | | | | | |
| G/G-C/C | 74 | 0.387 | 69 | 0.367 | 0.960 (0.626-1.474) | 0.853 | 0.867 (0.556-1.354) | 0. 941 |
| G/G-C/T | 27 | 0.141 | 27 | 0.144 | 1.056 (0.591-1.888) | 0.853 | 1.208 (0.650-2.244) | 0.860 |
| G/G-T/T | 3 | 0.016 | 0 | 0 | - | - | - | 0.529 |
| T/T-C/C | 16 | 0.084 | 15 | 0.080 | 0.980 (0.468-2.051) | 0.957 | 0.784 (0.356-1.730) | 0.556 |
| T/T-C/T | 9 | 0.047 | 4 | 0.021 | 0.453 (0.137-1.498) | 0.194 | 0.531 (0.146-1.930) | 0.558 |
| T/T-T/T | 9 | 0.047 | 0 | 0 | - | - | - | 0.365 |
| G/T-C/C | 33 | 0.173 | 35 | 0.186 | 1.140 (0.670-1.937) | 0.629 | 1.160 (0.666-2.021) | 0.091 |
| **G/T-C/T** | **4** | **0.021** | **19** | **0.101** | ***^b^*5.013 (1.531-18.121)**  **5.256 (1.753-15.760)^0.291^** | **0.005**  **0.003** | ***^b^*4.164 (1.232-15.343)**  **4.320  (1.390-13.428)^0.286^** | **0.035**  **0.011** |
| G/T-T/T | 2 | 0.010 | 0 | 0 | - | - | - | - |
